# Nanoparticles in Construction Materials and Other Applications, and Implications of Nanoparticle Use

**DOI:** 10.3390/ma12193052

**Published:** 2019-09-20

**Authors:** Abbas Mohajerani, Lucas Burnett, John V. Smith, Halenur Kurmus, John Milas, Arul Arulrajah, Suksun Horpibulsuk, Aeslina Abdul Kadir

**Affiliations:** 1 School of Engineering, RMIT University, Melbourne 3000, Australia; s3588855@student.rmit.edu.au (L.B.); john.smith2@rmit.edu.au (J.V.S.); s3432918@student.rmit.edu.au (H.K.); s3434124@student.rmit.edu.au (J.M.); 2 Department of Civil and Construction Engineering, Swinburne University of Technology, Victoria 3122, Australia; aarulrajah@swin.edu.au; 3 School of Civil Engineering and Center of Excellence in Innovation for Sustainable Infrastructure Development, Suranaree University of Technology, Nakhon Ratchasima 30000, Thailand; suksun@g.sut.ac.th; 4 Faculty of Civil and Environmental Engineering, Universiti Tun Hussein Onn Malaysia (UTHM), Batu Pahat 86400, Johor, Malaysia; aeslina@uthm.edu.my

**Keywords:** nanoparticles, materials, construction materials, risk assessment, health implications, environmental implications, sustainability

## Abstract

Nanoparticles are defined as ultrafine particles sized between 1 and 100 nanometres in diameter. In recent decades, there has been wide scientific research on the various uses of nanoparticles in construction, electronics, manufacturing, cosmetics, and medicine. The advantages of using nanoparticles in construction are immense, promising extraordinary physical and chemical properties for modified construction materials. Among the many different types of nanoparticles, titanium dioxide, carbon nanotubes, silica, copper, clay, and aluminium oxide are the most widely used nanoparticles in the construction sector. The promise of nanoparticles as observed in construction is reflected in other adoptive industries, driving the growth in demand and production quantity at an exorbitant rate. The objective of this study was to analyse the use of nanoparticles within the construction industry to exemplify the benefits of nanoparticle applications and to address the short-term and long-term effects of nanoparticles on the environment and human health within the microcosm of industry so that the findings may be generalised. The benefits of nanoparticle utilisation are demonstrated through specific applications in common materials, particularly in normal concrete, asphalt concrete, bricks, timber, and steel. In addition, the paper addresses the potential benefits and safety barriers for using nanomaterials, with consideration given to key areas of knowledge associated with exposure to nanoparticles that may have implications for health and environmental safety. The field of nanotechnology is considered rather young compared to established industries, thus limiting the time for research and risk analysis. Nevertheless, it is pertinent that research and regulation precede the widespread adoption of potentially harmful particles to mitigate undue risk.

**Highlights:**

- Nanotechnology Consumer Products Inventory (CPI) lists 1814 consumer products containing nanomaterials in 2015
- Nanoparticles valued for high surface area and low density
- Chronic TiO_2_ exposure yielded negative health effects in mice
- Ni, Co, and Pd potentially hazardous when absorbed through skin
- Nanoparticles listed as “Enabling Technology” in the EU
- Busy roads generate up to 9 kg of road particles per day, some nanoparticles (NPs)
- 97% of watershed sediment tested in Japan, France, and USA contained NPs

## 1. Introduction

The concept of nanotechnology was originally introduced by the famous physicist Richard Feynman in 1959, through his talk “There’s Plenty of Room at the Bottom”, which he delivered to an American Physical Society meeting at the California Institute of Technology. In the lecture, he discussed the possibility of using atoms as building particles to create nanosized products [1]. The ideas put forward by Feynman passed unnoticed until 1974 when Norio Taniguchi introduced the word “nanotechnology” at the International Conference on Production Engineering [2]. Taniguchi described the processes of creating semiconductor structures using various methods with nanometre precision. He introduced the “top-down” approach, which refers to the successive cutting or slicing of a bulk material to create nanosized particles. In 1986 Kim Eric Drexler developed the idea of nanotechnology and published a book titled “Engines of Creation: The Coming Era of Nanotechnology”. In the book, he proposed the idea of a nanoscale assembler, which would have the capacity and ability to build copies of itself and other things of distinctive complexity, known as molecular nanotechnology (MNT). He presented the “bottom-up” approach; the creation of material from molecular and atomic components—molecule by molecule, atom by atom. Following the ideas of the nanotechnological strategy, various inventions and discoveries were made in potential application areas of nanotechnology. A considerable intensification of the research and design took place and the publications on nanotechnology increased sharply.

The National Nanotechnology Initiative (2017) defines nanotechnology as “the understanding and control of matter at the nanoscale, at dimensions between approximately 1 and 100 nanometres, where unique phenomena enable novel applications” [3]. The prefix “nano” means 10^−9^ or one-billionth; hence, one nanometre is one billionth of a metre (International System of Units). A wide variety of production systems exist that are capable of manipulating and creating nanostructures to the desired shape, morphology, size, composition, and crystalline structure [4]. The two standard production approaches used are: “bottom-up” and “top-down”. In the bottom-up approach, complex structures are produced through the build-up of atoms or molecules. The common processes of the bottom-up approach comprise imprinting, laser trapping/tweezers, contact printing, and spinodal wetting/de-wetting [5]. While in the top-down approach, nanostructures are built from their macroscale counterparts without atomic level control. Common processes of the top-down approach include thin film deposition and growth, mechanical, electrochemical material removal, laser beam processing, and lithography processes [5].

Today, nanotechnology offers potential opportunities to create better materials with enhanced properties for use in various application areas. The atoms within nanoparticles are impeccably ordered; therefore, when the dimensions of a material are condensed from macro-size to micro-size (nano-size), substantial changes occur in the properties of the material [6,7]. The micro-dimensions of nanoparticles have led to a revolution of nanotechnology that has had a radical impact on the cosmetics, construction, electronics, manufacturing, and medical industries [8]. According to the Allied Market Research, in 2015, nanomaterials were valued at $14,741.6 million, and were expected to reach $55,016 million by 2022 [9]. The distinctive physical and chemical properties of nanoparticles allow the design of systems with high sensitivity, large surface areas, special surface effects, high functional density, catalytic effects, and high strain resistance [10]. In 2015, the Nanotechnology Consumer Products Inventory (CPI) listed “1814 consumer products from 6222 companies in 32 countries.” Silver was found to be the most regularly used nanomaterial while the health, medicine, manufacturing, electronics, and construction materials categories contained the most products [11,12,13,14]. Some of the applications of nanoparticles in construction are shown in Table 1.

In this review paper, the focus is on the current applications of nanoparticles in normal concrete, asphalt concrete, and bricks, as well as other construction materials. Modern construction materials for infrastructure and civil structures include concrete, steel, brick, and timber. With timber being a naturally occurring product that requires time and is limited by its material properties, the most common choices for large-scale projects are concrete and steel. Steel can be fabricated in large volume, to the design specification and is strong in tension but is expensive to process. Complementary to steel is concrete, which can also be mass-produced, is cheaper than steel to utilise, and is strong in compression. However, these materials are not without their flaws. Reinforced concrete and exposed steel need to be galvanised, painted, or have a cathodic protection system in place to prevent oxidation occurring and to protect the structural integrity of the material. Supplementary cementitious materials are generally added in the manufacturing of concrete as a replacement for more expensive clinker, and chemical admixtures are added for specific applications to enhance the properties of concrete [15]. These preventative measures increase the cost of steel and concrete, particularly if a great amount of material is required for the project. The amount of materials being consumed for construction projects around the world is exhausting the natural materials, and, as these materials are not reclaimable, such use is unsustainable. These are the issues that need to be considered when choosing materials for projects, which stem from the idea of whole life cycle costing in an effort to promote sustainable development and reduce the wastage of materials across the entire life of the structure [16]. The use of nanotechnology in the construction industry can solve many of these complications and can change the organisation and condition of certain construction processes. By combining nanoparticles and traditional building materials, exceptional properties can be achieved for the construction of long span and super high-rise systems [6].

The lower density and higher strength properties of nanomaterials provide a new impulse to the market growth of green high-tech [17]. Nanoparticles can assist in reducing the use of natural materials by improving the performance of construction materials and decreasing the consumption of energy. The use of nanoparticles in construction offers a cheaper, faster, and safer approach to the production of construction materials. In improving the technical properties of materials, the costs incurred during the life cycle of the nanomaterials can be reduced and a more rational approach to the use of raw materials in construction can be achieved [18]. Product durability and efficiency can be enhanced and higher performance levels in the production of raw materials can be achieved [17]. Nanotechnology has the potential to transform the construction sector into a period focused on environmental protection and innovation competitiveness, and, moreover, it has the capability to radically change our built environment by promising “more for less” [17]. According to the EPA USA, the amount of built space in the US will grow from approximately 300 billion square feet to 430 billion square feet in 30 years, where a significant source of solid waste will resume [19]. Therefore, it is necessary to employ different practices on a larger scale to steady the consumption of natural resources.

However, limited research exploring the plausible concern regarding the long-term effects of nanomaterials on the environment and human health is available. Adverse long-term effects may develop due to prolonged exposure to nanoparticles, as well as the use of products that incorporate nanoparticles [20]. Another major concern is the ability of the legal departments around the world to keep up with the ever-growing use of these nanoparticles, and to maintain consumer confidence with the products they are purchasing. This has called for some producers to list exactly what nanoparticles are being used as ingredients [20]. Furthermore, there have been additional concerns regarding the toxicity of the nanoparticles. Prior investigations have revealed that some nanoparticles can cause similar symptoms to that of asbestos fibres with reports of lungs becoming inflamed [21]. There have also been some further investigations that mention the potential for DNA damage during cancerous developments when exposed to nanoparticles. However, it could be argued that the level of human impact is dependent on the type of nanoparticle, volume, and concentration. Nevertheless, overall, it can be deduced that the concerns regarding the long-term effects from nanoparticles in the environment are of legitimate concern with further research required concerning the long-term human impact to promote the safe use of products containing nanoparticles [20].

## 2. Nanoparticle Definitions

### 2.1. Engineered Nanoparticles

Engineered nanoparticles are intentionally engineered through either bottom-up or top-down synthesis processes for application in a range of fields. These processes differ in approach, with the top-down synthesis gradually reducing larger structures in size to nanometre dimensions whilst the bottom-up synthesis involves constructing nanoparticles with individual atoms or molecules until the target size is achieved [22]. The top-down approach allows for higher production volumes, but due to the production methodology involving mechanical stress, violent shocks, and deformation, the final nanoparticle product may lack homogeneity in size and formation. The bottom-up approach allows for greater accuracy in nanometric state, allowing a multiplicity of nanoparticle structures, greater precision in molecular positioning, uniformity in grain size, and homogeneity of the final product, albeit at the cost of reduced production quantities [22].

Further classification may be made based on the synthesis process mechanism of formation, dividing the majority of engineered nanoparticles into three categories—chemical processes, mechanical processes, and physical processes [23]. Examples of these processes are given below (Table 2):

The list above outlines a few techniques for nanoparticle synthesis. A more extensive range of synthesis processes may be found in the British Standards document, Vocabulary Nanoparticles, 2005.

As nanoparticle viability has increased over several years, an upturn in both demand and production volume has occurred [24].

This trend is further evidenced in the empirical data reported in Piccinno et al. demonstrating a similar trending curve for volunteered production data [25]. Available data on nanoparticle production is scarcely available, with companies unwilling to provide information regarding production volumes or production capacities [25]. A snapshot of the 2012 nanoparticle production volumes was gathered through the voluntary provision of data, revealing that the most commonly produced nanoparticle is titanium dioxide (TiO_2_) at 10,000 t/y, with the production of other common nanoparticles, cerium oxide (CeO_2_), iron oxide (FeOx), aluminium oxide (AlOx), zinc oxide (ZnO), and carbon nanotubes (CNT) ranging between 100 and 1000 t/y [25].

### 2.2. Natural Nanoparticles

Nanoparticles occur naturally through a range of mechanisms commonly seen and can have a range of effects. Major producers of nanoparticle materials include volcanic eruptions, desert surfaces, and dust from cosmic bodies [26]. Volcanic eruptions produce solid, liquid, and gas by-products, with a large portion of particulate matter being suspended by escaping gas. As the gas cools, the particulate matter is suspended within accumulates and deposits. Deserts produce nanoparticles on mass, with an estimated 50% of all aerosols occurring in the troposphere originating from deserts, comprising a variety of materials represented in mass distributions. Furthermore, it was noted that toxic material, including cadmium, mercury, and polycyclic aromatic hydrocarbons (PAHs) occurred in an analysis undertaken in China and South Korea during a dust storm.

Nanoparticles can also occur in food products naturally, with a prime example being casein micelle, which is found within the milk of mammals. The process of milk generation involves the development of casein molecules. During this process, the casein protein assumes the micelle structure at a nanometre scale, existing within milk as a colloidal suspension [27]. Nanoparticles have also been found in foods that contain solid fats, with food items including chocolate, margarine, and lard demonstrating nanoscale platelets. These nanoscale platelets are generally 200 nm long, 80 nm wide and 20 nm thick, and act as the smallest microstructural elements of the colloidal fat crystal network [27].

Exposure to nanoparticles has not been a scenario that has resulted exclusively from the intentional engineering of nanoscale materials or from unintentional generation of nanoparticles through a variety of means, but also through food sources and from within the atmosphere.

### 2.3. Incidental Nanoparticles

The generation of incidental nanoparticles occurs through a variety of channels that are not immediately obvious in day-to-day life but can become apparent when identified and addressed. A clear example of incidental nanoparticles that occur in the built environment are those generated by cars through tyre material loss, brake pad material loss, exhaust emissions, and paint deterioration. Addressing the potential for negative health and environmental effects due to exposure to incidental nanoparticles is important to gauge the outcome of the interactions involving nanoparticles that are not immediately obvious. By identifying incidental nanoparticle and microparticle generation mechanisms, solutions can be proposed to address the issue. To highlight the oversight that may occur on a larger scale, tyre emissions can be assessed. It is generally assumed that fine particulate matter results from exhaust fumes and any larger particle emissions are due to tyre or brake pad erosion; however, it has been seen that exhaust and non-exhaust related by-products contribute near equally to Particulate Matter 10 micrometers (PM_10_) emissions, with non-exhaust contributions predicted to increase [28].

The volume of nano- and microparticles entering the environment due to motor vehicle erosion can be estimated in Australia over one year. In Australia, from 30 June 2015 to 30 June 2016 an estimated 18.2 million vehicles were registered, travelling an estimated cumulative 249,512 million kilometres, and averaging 13,719 kilometres per vehicle [29]. According to a publication released in 2014, the tread loss per 40,000 km in kilograms can vary from 0.73 kg to 1.21 kg, depending on the type of tyre and country under analysis [30]. By inferring a relationship between the US conditions and those in Australia based on similar manufacturers and production standards, an assumed value of 0.88 kg/40,000 km may be assumed, or 0.022 g/km, [30]. Over the course of one year in Australia due to tyre erosion alone, 5489.3 tonnes of rubber are released as fine particles becoming airborne or remaining on the road surface. Of the 5489.3 tonnes of rubber eroded over the course of 2016, up to 10% occurs as airborne PM_10_ particles or 549 tonnes of fine airborne particles. Due to the limitations in previous studies, the exact Particle Size Distribution (PSD) of tyres has not been identified with some researchers finding bimodal size distributions in coarse and fine particles and others unimodal size distributions in coarse particles only. However, a significant part of tyre and brake wear PM_10_ lies in the fine size fraction [28]. The figures presented above are subject to a range of factors and a dramatic variation in particle size and material loss can be attributed to altered conditions including the mode of driving, speed, temperature, type of car, and type of tyre.

## 3. Methods

This paper attempts to comprehensively review the research into the use of nanomaterials in construction materials and identify further areas of study required for wide-scale industry adoption. The literature for review has been selected on the basis of summarizing the current state of nanotechnology research, while highlighting key areas for future research. The literature reviewed has been sourced from peer-reviewed journals, textbooks, and government publications. Particular attention has been paid to research on environmental and human health impacts, recycling of waste nanomaterials, and other potential issues preventing industry adoption of nanotechnology in construction materials.

## 4. Nanoparticles in Construction Materials

### 4.1. Concrete

Concrete has been used as a construction material for many years. The primary components of concrete used in modern construction include Portland cement-based binders, water, and coarse and fine aggregate. The binders are made from Portland clinker ground together with a little calcium sulphate and can also contain fine mineral powders, such as pozzolan (usually volcanic ash), limestone, granulated blast furnace slag, and fly ash (commonly from coal-burning power plants) [15]. To modify the properties of concrete for particular applications, chemical admixtures, such as air-entraining agents and superplasticisers, are added in small quantities [31]. It is important to develop these changes to convention as the durability and serviceability of concrete structures and surfaces are constantly tested under the exposure of different weather conditions. The durability of concrete depends on the connection interfaces between the voids, the aggregates, and the cement paste [32]. Therefore, nanomaterials with properties, such as durability and strength, are of particular interest in the production of concrete [10].

**Figure 1:**
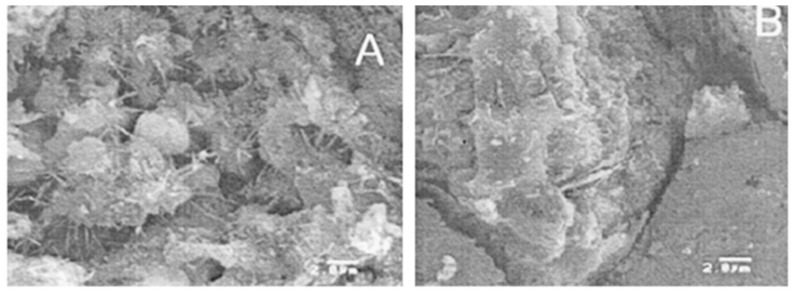
SEM micrographs of plain (**A**) and nano-silica modified cement paste (**B**) [32].

In conventional concrete, silica (SiO_2_) is present as part of a standardised mix. However, in recent studies, it was found that the use of nano-silica (NS) in concrete and cement pastes improved the particle packing in both materials [33]. NS acts as a nanofiller for the calcium silicate hydrate (Ca–Si–H) particles in the cement and acts as a strong binding agent, thereby increasing the cohesion between the cement and the aggregate. The rate of cement hydration has also increased, which effectively reduces the setting time, dormant period, and increases the early strength. Nano-silica also reduces the porosity of the concrete, reducing the ability of water and other elements to penetrate the concrete, which avoids the potential for concrete degradation [32]. Figure 1 below illustrates scanning electron microscope (SEM) micrographs of a plain cement paste and a nano-silica modified cement paste.

Another nanoparticle used in concrete is nano-titania (TiO_2_), which has been produced in abundance due to its anticorrosive, stable, and photo-catalytic properties [34]. The photo-catalytic activity of TiO_2_ is due to the high surface area of the particles; therefore, when added in concrete the concrete becomes self-cleaning, self-disinfecting, and environmental pollution cleansing. In the presence of light, TiO_2_ breaks down the organic pollutants and dirt on the surface of the concrete into harmless water and CO_2_; the products of the catalytic reaction are then easily removed by simple rinsing or rain.

At present, carbon nanotubes (CNT) are being incorporated in concrete as a nanofiller due to CNTs high surface area and extraordinary mechanical properties. Konsta-Gdoutos et al. proved through scanning electron microscopy (SEM) that the pore spaces in concrete could be filled with CNTs more effectively [35]. The addition of CNTs to concrete can make concrete impenetrable to salts and water, therefore significantly enhancing the durability properties of concrete [35].

### 4.2. Asphalt Concrete

Asphalt concrete (asphalt) is a composite material used to cover the surface of roads, driveways, and airport runways. Its ability to withstand high volumes of traffic load as a surface cover for the road foundation makes asphalt an invaluable construction material. However, it is not without its limitations. Currently, it is difficult to meet the design requirements for extreme temperatures. The resistance of asphalt to high and low temperatures is weak, which results in the asphalt melting or cracking [36]. However, to account for the extreme temperatures, the modification of asphalt’s material properties of ductility and elasticity can be changed with the addition of nanoparticles with the most popular modifiers being styrene–butadiene–styrene (SBS), styrene–butadiene–rubber (SBR), and polyethylene (PE) [37].

Fortunately, the list of modifiers is not limited to those mentioned above. Nano-aluminium oxide (Al_2_O_3_) has been tested in asphalt cement to check the impact of the modifier on the properties of asphalt cement. A 5% inclusion of Al_2_O_3_ in the asphalt mix design showed strong resistance to high temperatures [38]. This type of modifier offers another alternative to the list of modifiers currently used for asphalt. With these and other nanoparticles acting as modifiers to asphalt, it has been deemed that the integration of nanoparticles in asphalt has greatly improved the serviceability of the material [39,40].

Moisture damage has been a big issue in asphalt pavements lately and is considered to be a big problem around the world. It may cause failure of the hot mix asphalt layers due to the infiltration of water in the pavement structure. This can cause a loss of stiffness, durability, and strength [41,42]. In a study, the effect of zycosoil as an anti-strip agent was evaluated to investigate the properties of asphalt concrete when prepared with zycosoil [43]. The results show an increase in the fatigue life due to the increase of the filler and the decrease of air voids in the asphalt mixture due to the aggregate coverage of zycosoil. Moreover, better compaction results of the asphalt mixture are achieved due to the modification of the aggregate surface [41].

### 4.3. Bricks

Bricks have been used as a construction material for many years. The current composition of bricks consists of 50% clay but no greater than 80% clay, with the remainder of the earth brick made up of sand and other granular materials. With these materials bound together under high temperatures, a good compressive strength material is formed which makes the earth brick an exceptional domestic construction material [44,45].

However, existing earth bricks do not demonstrate good compressive strength. Niroumand et al. conducted compressive strength tests to check the influence of nano-clays on earth bricks [46]. Nano-clays are considered to be layered mineral silicates of nanoparticles and the use of varying nanoparticles in bricks is dependent on the chemical makeup of the brick. The results show that a 5% inclusion of nano-clay can develop a compressive strength 4.8 times that of normal clay bricks [46]. Over the life of the brick material, the nano-clay modifier prevails as the more sustainable material when compared against normal clay bricks.

Stefanidou and Karazou tested the effectiveness of different protective coatings on bricks and compared the physical properties of each solution [47]. Linseed oil, silane/siloxane, and alkosiloxane modified with 1%–1.5% silica nanoparticles were tested. The results show that alkosiloxane and silica nanoparticles prove to be the most effective way to protect bricks. The treated bricks show high resistance to water uptake and a significant improvement in terms of durability.

### 4.4. Mortar

The binder between the bricks and other masonry construction materials has also changed the chemical composition in recent years. Like the clay bricks, the mortar binder has similar permeability properties and allows for the quick evaporation of moisture from the material. However, the water that permeates within the binder can cause material loss, resulting in a reduction in the composite strength of the binder. If this binder were allowed to degrade, then the surrounding brick material would soon follow. Therefore, to be able to use mortar in water-rich areas, it is necessary for the mortar to be coated with organic oils or oil/wax additives.

However, nowadays, nano-clays are used as an epoxy coating as part of the sustainable initiative to conserve energy, including the embodied energies used to repair/replace material [47]. Through extensive experimental tests, the results showed an increase in the surface roughness and a reduction in surface free energy. Furthermore, with the combination of silica nanoparticles, a higher efficiency and higher permeability solution was achieved.

### 4.5. Timber

Timber is a natural building material that offers many advantages and excellent performance. It is a sustainable building material that stores carbon dioxide, necessitates smaller amounts of energy to manufacture, and is renewable. It is cost effective compared to other building material choices, provides strength, and thermal performance, and allows designers to create durable, fire resistant, and strong timber constructions due to the predictable performance and measurable nature of the materials. However, timber is fairly sensitive to biological attacks; therefore, coatings are necessary to enhance the surface characteristics, improve the durability, and protect the internal structure of the material. Lately, alumina and silica nanoparticles (SiO_2_) have been incorporated with hydrophobic polymers to develop a timber coating [17]. The coating is an invisible layer formed on the surface of the timber with outstanding water, oil, dirt, algae, and dust repellent properties. The coating further provides UV protection and retains the quality of the surface [48].

One fabrication of wood is medium density fibreboard (MDF), which is an engineered wood product composed of a synthetic resin combined with fine lingo cellulosic fibres that is subjected to pressure and heat to form panels [49]. The method adopted to form the panels is considered costly, therefore decreasing the pressing time will decrease the production costs and increase the production capacity. Kumar et al. studied the effect of nano-aluminium oxide (Al_2_O_3_) on the heat transfer process of MDF during hot pressing. The data obtained show an improvement in the mechanical and physical properties of MDF panels due to the enhancement in the heat transfer that helped the curing of urea formaldehyde (UF) throughout the mat [49].

Goffredo et al. studied the potential of titanium dioxide (TiO_2_) due to its photoactivity under UV illumination as a biocide and its antifungal and antibacterial use on wood surfaces [50]. The results show that the nano-treatment moderately inhibits the growth of Aspergillus niger hyphae and the extent of germinated conidia covering the wood surfaces. However, it was concluded that further studies were required to analyse the efficiency of TiO_2_ and metallic nanoparticles to inhibit fungal development.

### 4.6. Steel

Steel products are one of the most extensively used building materials in construction due to its durable nature; holding the highest strength to weight ratio compared to other building materials. Furthermore, steel is fire resistant; therefore, during a fire it will not burn and provide fuel. It is the prominent construction material for sustainability, as it can continuously be recycled since the process does not have a damaging effect on its properties. However, stronger steel types are being produced by adding nanoparticles into paints for steel coating when used as reinforcement bars for concrete construction. These bars are known as micro-composite multi-structural formable (MMFX) steel and are preferred over conventional steel due to their corrosion-resistance and durable properties [17]. With conventional steel, surface unevenness leads to stress risers, and, therefore, fatigue cracking; however, with the use of nanoparticles as a modifier, surface unevenness can be reduced, and, therefore, cracking [18]. Furthermore, studies have shown that the effects of hydrogen embrittlement and the inter-granular cementite phase are reduced through the improvement of the steel microstructure [17].

Hegazy et al. studied the influence of colloidal copper nanoparticles as a modifier for steel anti-corrosion paints [51]. A colloidal dispersion solution was prepared of copper nanoparticles using the chemical reduction method of copper (II) chloride (CuCI_2_). Several tests were conducted to investigate the properties of the modified coating on the carbon steel. The steel anti-corrosion coating shows a maximum inhibition efficiency when exposed to 0.5wt% copper nanoparticles solution. The results show that the modified coating provides further corrosion protection and good coverage of the carbon steel.

## 5. Environmental, Social, and Economic Benefits

The development and growth of nanotechnology can potentially improve the properties of construction materials, and, therefore, support sustainability. There is a huge amount of waste concrete around the globe with approximately 317 MT of concrete waste in the USA, 510 MT in Europe, and 239 MT of waste in China during the period of 2009–2010 [52]. This level of wastage is considered to be unacceptable, especially with the depletion of the world’s natural resources beyond the industrial revolution, and this has led to a call for changes to the environmental management plans of construction sites, which now state that the materials on the site should be stockpiled and separated so that they may be reused and recycled for other construction projects. From these recycled products, various sized aggregates using sieves can then be used in future projects to improve the sustainability of structures and reduce the overall embodied energy [52]. Furthermore, the construction industry is always looking at other ways to improve the environmental and economical sustainability, and, even though using nanoparticles in concrete has improved constructability and resulted in greatly improved construction times, there is the issue of recycling these nanoparticles along with the health hazards associated with them to workers in demolition/construction and members of the communities in the area [53].

The recycling of concrete with nanoparticles has been investigated recently and it has been found to possess higher compressive strength than that of typical recycled normal concrete [54]. One of the main concerns regarding the recycling of concrete without nanoparticles is that it is inferior to normally prepared concrete with regards to durability and mechanical properties. This is an issue when attempting to use recycled concrete for large-scale infrastructure and projects, which invite a higher risk, and, hence, may deter the selection of the recycled concrete. However, it has been found that the addition of nanoparticles to the recycled normal concrete can produce mechanical properties of the level of fresh normal concrete [54]. The strength and the microstructure are improved but the workability of concrete is reduced with the addition of nanoparticles. It has also been determined that recycled concrete with nanoparticles can achieve comparable compressive strength to that of fresh normal concrete after 28 days, when the percentage by mass of nano-silica is 3% by mass [54]. The current research available, however, is limited, and more work should be conducted to investigate the dynamic mechanical properties of the effects of nanoparticles with the recycled concrete and how this compares against fresh normal concrete in terms of impact loading [54].

## 6. Risk Assessment and Analysis

In recent years there has been a significant increase in the use of nanostructures in a variety of construction materials including concrete, steel, bricks, timber, asphalt concrete, and mortar. However, limited scientific evidence has been gathered that deny or support the implementation of nanotechnology in the construction industry. There are major potential risks that need to be considered at all stages of nanotechnology. Therefore, the following sections review the components required to carry out a risk assessment on nanomaterials. The key aspects analysed and addressed include a toxicological analysis, and short-term and long-term exposure risks of nanomaterials on the environment and human health during the life cycle of the materials, and the risks associated with the disposal of nanomaterials.

### 6.1. Toxicological Analysis

One of the main complications of nanotechnology is the identification and characterisation of nanomaterials and their aggregates [55]. When materials decrease from macro size to micro size, substantial changes occur in their physical and chemical properties. Therefore, understanding their properties is a critical factor in determining the potential toxicological hazards and their exposure to the environment [56]. However, due to the continuous development, diversity, and complexity of nanomaterials, the chemical identification and characterization can be difficult. A comprehensive spectrum of the physical and chemical properties needs to be characterized adequately and appropriately, including but not limited to the structure, boiling point, melting point, composition, reactivity, water solubility, stability, molecular weight, octanol–water partition coefficient, and vapour pressure [4]. However, the properties listed above may be insufficient in characterizing nanomaterials due to the lack of existing methods and information for hazard and exposure evaluation. More insightful investigations and broad studies on the development of well-characterised nanomaterials are essential [56].

### 6.2. Epidemiological Analysis

Epidemiological analysis entails a review of the determinants of the health and disease conditions in a general population. In depth epidemiological analysis must be undertaken to determine the relationships between the three classes of nanoparticles—engineered nanoparticles, natural nanoparticles, and incidental nanoparticles as seen in tyre erosion through shear stress mechanisms and any carcinogenic response [28,57].

Regarding engineered nanoparticles, it has been concluded that there is inadequate epidemiological analysis to conclude a causal relationship between exposure to particles and lung cancer risk [58]. An epidemiological survey of workers exposed to carbon black and TiO_2_ with a quoted particle diameter range of 1 to 500 nm, was completed and summarised to gauge the relationship between exposure and cancer risk. With the exception of a large population of carbon black workers in Germany where a doubling of cancer risk was noted, the findings proved to be inconsistent and the International Agency for Research on Cancer (IARC) deemed the evaluation to yield “inadequate evidence in humans for the carcinogenicity” [58]. Due to the inconsistent nature of the results obtained, further study is required. However, the results may vary drastically due to the properties of the nanoparticles under study, as epidemiological analysis includes particles ranging from 1–100 nm, of varying material—both reactive and inert—and varying drastically in terms of the material density or surface area and particle solubility.

### 6.3. Life Cycle Exposure Pathway

As the development and use of nanomaterials increase, their exposure risk to the environment also increases, which intensifies the possibility for detrimental effects on the environmental and human health [59]. Manufactured and processed nanomaterials may enter the environment directly and/or indirectly during all stages of their life cycle. This may be through material and chemical manufacturing processes, the release of nanomaterials during use on a construction site, consumer use, and release resulting from the disposal of nanomaterials. Once nanomaterials are released into the environment they may experience distinctive chemical, biological, and physical transformations, which may result in a change in their properties [8].

#### 6.3.1. Manufacturing of Nanomaterial

The manufacturing/synthesis process of nanoscale materials is the primary exposure route to nanomaterials. Most processes are performed in closed reaction chambers; therefore, exposure through inhalation is limited during the manufacturing process. However, there are still considerable sources for environmental release during and after the manufacturing process including the evacuation of wastes from cleanout operations, losses during spray drying, nanomaterial handling, filter residuals, and emissions from filters [4]. Tsai et al. found a substantial release of nanoparticles during the handling of dry powders containing nanoparticles inside laboratory fume hoods [60]. The nanoparticles escaped from the fume hood into the laboratory environment and into the researcher’s breathing zone. Work practices, hood design, quantity and type of nanomaterial being handled, hood operation, and room conditions were found to affect the degree of nanoparticle release [61]. Most nanomaterial exposures are in the form of agglomerates/aggregates; therefore, when particle sizes are measured, analysers are unable to reveal the agglomeration state and the degree to which an agglomerate can separate into several more units in the lung fluid [62]. This is due to the limited number of publications on the field of occupational exposure to nanomaterials.

#### 6.3.2. Use in Construction Site

Particles on worksites during the periods of construction can have prolonged exposure in the atmosphere without implementing appropriate chemical management policies. With the example of dust on worksites, the adverse health effects on workers through dust inhalation can result in workers taking sick leave and adding unnecessary costs to the projects. An example of a management plan may involve the watering down of the areas to be worked, to reduce the risk of dust inhalation to workers and the nearby public. With the inclusion of nanoparticles in concrete construction and recycling, a new hazard is introduced, and the risk management plans need to be accommodating of this change [52]. When preparing nano-silica for use with cement, it can either be prepared dry or wet [63]. When preparing dry, the use of superplasticisers is recommended, and when preparing wet, the nano-silica is dispersed in water and then added to the cement mixture. This wet method of adding the nano-silica is a far more effective process for the commercial production of the concrete and reduces the risk of an adverse impact on humans, as the nanoparticles are suspended in solution, as opposed to being exposed to the atmosphere during the dry mix [63]. The manual application of spray coatings on construction material poses a very high exposure risk for workers due to the high particle concentration, compared to composites, which are generally bound in the composite matrix [4]. It should be noted that further research should be conducted on the specific nanoparticles and ultra-fine particles as there is limited health and safety risk information and management, with minimal regulatory guidelines [52]. These processes may be similar to the cases of asbestos used in the construction industry.

Although asbestos is now known to be carcinogenic, previously it was used extensively as a construction material due to its high resistance to temperature and strong insulation properties [64]. There are still structures around the world, from domestic housing to underground services that exist and contain asbestos within them that have yet to be removed. The main issue with asbestos is its porous form, which can induce a variety of cancers in exposed workers [64]. There are however appropriate procedures with the management of the chemical that involve appropriate training, appropriate PPE (Personal Protective Equipment), and watering down of the asbestos areas, similar to the dust management policies for worksites. However, it was only after all the reported health incidents with asbestos occurred that such procedures for removal were developed to reduce the risk to workers and the public in the vicinity. The majority of Europe, as well as Australia and New Zealand have now banned the use of asbestos due to its severe health risks, but some countries remain avid producers and users of the material [65]. With the limited research knowledge and management policies that have occurred in the past with asbestos, it is safe to recommend that further testing and research development be undertaken prior to the recycling of concrete with nanoparticles.

#### 6.3.3. Demolition

The demolition of a structure poses high environmental exposure risks due to the release of nanomaterials. It is critical to remove and dispose of any hazardous material before undertaking the demolition process with the use of explosives or heavy disruption machines. Although coatings are relatively easy to remove, composite structures, such as concrete modified with nanoparticles are almost impossible to separate [59]. Therefore, during the demolition stage, the release of nanomaterials into the environment can be uncontrollable, and, hence, why strict standard demolition procedures need to be applied [66]. The crushed solid nanomaterial waste from the demolition activity should then be transported to disposal sites, which may be a prevalent route for environmental exposure.

#### 6.3.4. Recycling

Recycling waste containing nanomaterials is a growing concern due to the risk of potential nanoparticles being released into the atmosphere and environment; it is the specific recycling method used that determines the risk of exposure to nanoparticles [67]. When modified construction materials are recycled, the particles may form new agglomerates or remain individually isolated. Preferably, construction materials engineered with nanoparticles should be formed in a way that allows the nanoparticles to be separated from the modified material and re-used easily [68]. Mykonkaya et al. demonstrated a concept of reversible control over colloid stability applied to typical nanoparticle systems [68].

#### 6.3.5. Long-Term Release

Whether natural or artificial, the deterioration, abrasion, and damage of buildings during their lifetime can cause nanomaterials to be released into the environment. Natural disasters, such as floods, heavy rainfall, or storms, can impose damage on structures, stimulating the leaching or dissolution of nanomaterials into soils and natural waterways. Furthermore, a fire can release nanomaterials into the atmosphere. Due to the current analytical limitations, it is very difficult and challenging to characterise the release of nanomaterials on a long-term basis.

## 7. Health Implications

Exposure to nanoparticles arises in a multitude of ways, during synthesis in controlled environments, application in industry, deposition from construction material during lifetime and recycling of nanoparticle impregnated materials. During the specific application of nanoparticles, exposure can result in the inhalation of nanoparticles or absorption through direct contact with unprotected skin. The effects of this exposure are poorly understood requiring research to expand on the interactions between organic materials and nanoparticles. Studies on the inhalation of various common nanoparticles by mice have yielded insights into the potential adverse effects of exposure; however, generalisation of the results is limited by the experimental subject. Some of the health implications of nanoparticles to human organs are summarized in Table 3.

### 7.1. Pulmonary Exposure

Inhalation is a primary mode of transfer for nanoparticles into the body, raising concerns of pulmonary carcinogenic reactions similar to that caused by asbestos where inhaled minute crystalline fibres induce asbestosis, lung cancer, pleural fibrosis, and mesothelioma [78]. Due to the lack of understanding of the health risks associated with asbestosis, workers were exposed to the carcinogenic fibres for many years prior to the introduction of legislation. Inhalation of particles on construction sites has since been identified as a health and safety concern, with measures implemented to reduce inhaled dust via watering down.

Diesel engine exhaust is classified by the IARC as a carcinogen, containing fine particles of transition metals and polycyclic aromatic hydrocarbons (PAH) [79]. Particle size distribution analysis of diesel fuel exhaust demonstrates that up to 53%–84% of particles are within the nanoparticle size range. Deposition results demonstrate that 70% of particles under 0.1 micrometres, and 45%–70% of particles from 0.1 to 1.0 micrometres deposit in the alveolar–interstitial level [80]. A comparison of the particle sizes under the nanoparticle definition raises potential concerns for similar deposition traits, however reactions to alternative particles may yield different results. It is of note that PAH interaction with nanoparticles alters the oxidative capacity of the material, inducing oxidative stress within the body, a condition not replicated in many nanoparticle exposures [80].

Potential epidemiological links may be made by expanding on and generalising from rat and mice studies on inhalation related carcinogenic reactions. The validity of studies completed on mice may lack relevance when analysing a disease relationship because the networks linking genes and disease have a probability of differing [81]. Furthermore, a lack of reproducibility casts doubts on the results, although this may be attributable to bias in reporting, improper data analysis of key factors, and inaccurate or incomplete experimental condition descriptions [82]. In lieu of long-term analysis using human subjects regarding common engineered nanoparticles, correlations with mice and rat data must be utilized.

Reaction to nanoparticles is predicated on the type and volume exposed as microscale interactions within the body may vary vastly between common nanoparticles. One of the most common nanoparticles used within industry is titanium dioxide (TiO_2_). The particle is valued for its larger surface to volume ratio and is used in the food, clothing, electronics, medicinal, agricultural, and construction industries [83]. Research into chronic nasal exposure in male mice was undertaken to relate the results to humans, such that the pulmonary response to TiO_2_ may be quantified. Male mice were exposed to 1.25, 2.5, or 5 mg/kg TiO_2_ nanoparticles for a length of nine months via nasal installation, to ascertain the effects of chronic exposure. The results demonstrate a reduction in body weight, infiltration of inflammatory cells, and tumorigenesis in mouse lung tissue, with concurrent biochemical dysfunction evident [83]. However, nasal instillation, as utilised as the exposure mode, does not reflect the expected occupational exposure, and, therefore, may not capture the full effects.

Similar studies were undertaken using nickel hydroxide, exposing mice over varying time periods to Ni(OH)_2_ particles of 40 nm count median diameter via aerosol inhalation. Aerosol inhalation replicates occupational exposure more accurately, reduces the stress levels of mice due to limited necessary restraint, and provides uniform exposure to all the mice in the experiment thereby providing less extraneous variables to alter the results [84]. The findings show that with exposure to Ni(OH)_2_ nanoparticles lung inflammation ensues, with a varied reaction dependent on the exposure duration. Inflammation was evidenced by increased polymorphonuclear leukocytes (PMN), macrophages, and lymphocytes; however, damage was deemed reversible due to the lack of severe inflammation indicators [84].

Exact relationships between inhaled nanoparticles and disease or tumorigenesis have not been ascertained; however, data does suggest there may be a link. Due to the uncertainty surrounding the potential adverse health effects, further research is required. As was not seen in industry regarding asbestos, legislation, safety recommendations, and regulation must preface the widespread use of nanoparticles to prevent potential disease [78].

### 7.2. Dermal Exposure

Nanoparticles may be absorbed through the skin, thus gaining entry to the body. Four modes—intercellular, transcellular, and trans-appendageal through either hair follicles or sweat glands—of dermal absorption have been identified, which are dependent on the properties of the specific nanoparticles in question [85]. Absorption through the derma is often overlooked in favour of inhalation exposure studies, and the assessed risk is low due to the perception of less permeability via the skin [85]. In a study using porcine skin, polystyrene nanoparticles were distributed using vertical diffusion cells for 0.5, 1, and 2 h intervals. Surface imaging revealed an accumulation of smaller nanoparticles in the follicular openings. Studies into the effects of mechanical flexion have demonstrated that movement enables the transition of micrometre particles from the outer dermis into the lower dermal layers [85]. However, as the information gathered from dermal absorption studies is contradictory, more studies are required to clarify the interactions between the skin and nanoparticles. Further experimentation must be pursued due to evidence suggesting that nanoparticles may pass through the skin at sites of flexure, wounds, and lesions [85].

In a 2015 review of existing dermal absorption research, a variety of findings were compiled. Most notable among these is the particle size influence on absorption, with nanoparticles ≤4 nm being able to penetrate/permeate intact skin, nanoparticles 4–20 nm potentially able to penetrate/permeate intact or damaged skin, nanoparticles 21–45 nm only able to penetrate/permeate damaged skin, and nanoparticles ≥45 nm unable to penetrate/permeate the skin [86]. Furthermore, specific nanoparticle dermal interactions were noted, with major cases listed:

- TiO_2_ and ZnO nanoparticles were unable to pass through skin rendering them safe to dermal exposure.
- Ag nanoparticles can penetrate the skin but their ability to permeate is undetermined. Their use in medical dressings may result in high skin absorption, potentially affecting internal organs.
- Au nanoparticles can penetrate the skin, but their permeability is undetermined. Concern in terms of being a hazardous material is low as gold is a noble metal, and, thus, non-toxic to human health.
- Ni, Co, and Pd are considered more hazardous due to the high release of ions in the body [86].

Specific hazard awareness for nanoparticles needs to be elaborated so that occupational risks may be further reduced. This is of particular necessity due to the underestimated risk associated with the absorption of nanoparticles through the skin [86].

## 8. Environmental Implications

With the rise of nanoparticle applications and demands from previously unrelated industries, the exposure and production of nanoparticles has risen. Thus, through transport, erosion, washing, disposal, and erosion of nanoparticle enriched products, nanoparticles will find their way into ecosystems [87]. The impact of the introduction of nanoparticles into marine and land environments is currently ambiguous, with the direct results of nanoparticle exposure unclear. Materials that were previously deemed safe to environments must be reassessed at a nanoscale dimension due to the previously unforeseen interactions that nanoparticles experience [87]. Thus, it is pertinent to consider the implications of nanoparticles in the environment and judge the cost-benefits of their use in industry.

One of the key issues with nanoparticles is the way that interactions occur involving materials that were previously considered to be nontoxic. Specifically, although silver has hitherto been considered nontoxic, evidence suggests that exposure to silver nanoparticles in the embryotic stage of zebrafish can trigger development abnormality and or death at concentrations at or above 0.19 nanomolar [87]. Furthermore, non-marine examples provide evidence of potential issues arising from environmental nanoparticle interactions, with silver nanoparticles disrupting the seed growth of a variety of plants, causing silver accumulation in shoots, and bioaccumulation in green algae, rag worms, and gastropods [87].

Nanoparticles are released to the environment through a multitude of channels, both intentional and unintentional, via atmospheric emissions, and effluent, or through remediation of contaminated land or water [88]. Products enriched with nanoparticles comprise a proportion of the nanoparticles being deposited in the environment, and, thus, it may be postulated that as the use of nanoparticles increases, the same relationship will hold true for environmental nanoparticles [88]. Engineered nanoparticles are the primary concern of risk analysis regarding nanoparticles, but it is integral that incidental nanoparticles not be overlooked. Tyre rubber wear particles and brake pad wear particles are commonly neglected, despite contributing a significant portion of particulate emissions annually. A busy road with 25,000 cars travelling daily will generate up to 9 kg of particulate matter per kilometre of road, with no remediation [89]. This has led to the deposition of automotive related particulate matter deposition in streams and lakes. A study of 149 sediment samples collected from watersheds in the USA, France, and Japan demonstrated a 97% detection rate, with values ranging from <14–5800 ppm in dry weight. An average value of 1000 ppm and mean of 440 ppm demonstrates a vast difference in concentration with France, on average, registering five times the concentration of particles than the USA or Japan. However, this is theorised to be due to the silt and clay content of the Seine River, France [90].

## 9. Regulation

As nanoparticle research continues and applications for nanoparticles in common materials develops, so too must regulation reflecting the relevant health considerations for all periods of exposure. The predominant method of exposure to engineered nanoparticles is during synthesis and utilisation, during which workers are exposed to the risks of inhalation, consumption, and contact exposure. In addition to synthesis and implementation exposure, the general population may be exposed during the lifetime of products containing nanomaterials. Such regulation is key in limiting potential harmful effects to people, fauna, and marine environments, as evidence suggests that such demographics are at risk. This is evidenced by a study of fine particulate matter in the lungs of asthmatic children, with results showing that the majority of fine particulate matter, which is known to have an adverse effect on lung function, present in the lungs of the subjects of the study was man-made carbon nanotubes [91]. Marine exposure to nanoparticles has a two-fold risk profile with marine organisms breathing water impregnated with nanoparticles, causing adverse effects in these ecological systems, and increasing the exposure of humans to nanoparticles via the consumption of marine food sources exposed to nanoparticles. It can be seen that the effects of nanomaterials in marine environments is poorly understood, with researchers looking into the impacts on specific wildlife. The research undertaken on the toxicity of silver nanoparticles in zebrafish demonstrate a size dependent reaction, with finer particulate matter (20 nm) occurring in significantly higher concentrations in the gills than larger particulate matter (120 nm) [92]. Analysis of the intestines demonstrated similar concentrations of both 20 nm and 120 nm diameter particles in exposed fish, with intestinal particulate materials at parity for fish following a seven-day depuration period [92]. It is predicted that, annually, in the United States alone, due to their application in a wide range of consumer items, over 60 tonnes of silver nanoparticles reach the surface waters. Due to the startling revelations of research into the extent of exposure to nanoparticles and the unknown effects of exposure to both humans and biological systems, regulation must be introduced to curb and ensure that everything practicable is being done to limit the potential hazard development.

### 9.1. United States

The United States EPA defines nanomaterials as a diverse class of substances that has structural components smaller than 100 nanometres. As such, nanoparticles are a subset of this definition, possessing two dimensions of between 1 and 100 nanometres and referred to by the EPA as ultrafine particles (UFPs) [51]. The US is regulating nanoscale materials under the Toxic Substances Control Act (TSCA) to ensure that the manufacture and use of nanoscale materials do not pose unreasonable risks to human health and the environment [86].

To develop a further understanding of nanoscale materials, the EPA introduced a rule stipulating the requirement of persons who manufacture, process or import a reportable substance under the TSCA to declare specific chemical identity, production volume, methods of manufacture, processing, use, exposure and release information, and available health and safety data. All persons responsible for the manufacture, process or import of nanomaterials within the three years previous to the rulings end date were required to declare. Following the temporary rule lapsing on the 12 May 2017, a final rule with provisions regarding the reporting of information relating to nanoscale materials became effective on 12 May 2017 [93].

Under the TCSA, manufacturers of new substances are required to provide information to the EPA for review prior to the manufacture and introduction to the market. Following the review, the EPA may take action to ensure that chemicals that may pose unreasonable risk to human health or the environment are controlled [94]. Since 2005, the EPA, under the TSCA ruling, has received and reviewed over 160 new chemicals within the nanoscale materials bracket [89]. This information has allowed the EPA to make informed decisions on matters surrounding nanoparticles, and, where deemed necessary, limiting their application, requiring the use of personal protective equipment, introducing engineered controls, limiting environmental releases, and requiring the generation of both health and environmental effects data. Furthermore, the EPA permits the limited manufacture of nanoscale materials through the TCSA or through specific regulatory exemptions, provided exposure is controlled against all practicable risks.

### 9.2. Australia

The Australian body for workplace safety, Safe Work Australia, has identified that engineered nanoparticles fall under the same legislation governing technologies, chemicals, substances, and materials, and requires the associated risks to be addressed via elimination, minimisation, or communication. However, this places the burden of responsibility for worker safety regarding nanomaterials in the hands of managers, who may have little to no knowledge of the potential risks of nanomaterials, and which is exacerbated by the recent growth in the application and popularity of nanomaterials [85]. Safe Work Australia released recommendations regarding nanomaterial emission and exposure management, to be adopted within workplaces. Such recommendations are only applicable to the workplace, and any exposure falling outside the bounds of a worksite is not governed by Safe Work Australia, but rather the Department of Health National Industrial Chemicals Notification and Assessment Scheme.

### 9.3. Europe

In 2012, the European Commission deemed that the regulations regarding chemical releases into the environment, in both commercial and household waste applications, can apply to nanomaterials [85]. However, the regulations contain gaps or clauses that may be subject to challenge. In 2011, the EU adopted a broad definition of nanomaterials to include a variety of materials in different modes, including particulate and colloidal suspensions, such that the legislation made by the body may be applicable in all practicable scenarios. The adoption of the recommendation for nanomaterials laid the foundation for stronger legislation, and, as such, regulation to ensure that undue risk is not taken with materials that may have the potential to be hazardous. As seen in the EU, one of the greater challenges affecting the efficacy of nanomaterial legislation is identification of the presence of nanomaterials in products to determine whether the nanomaterial definition as recommended by the EU is applicable, and, as such, is specific legislation applicable [95]. In respect of the nanomaterial legislation, it can be seen that all the legislation written specifically addressing nanomaterials is only applicable to the application for which it was written, whereby legislation with language directing the application of nanoparticles in cosmetics is not applicable to food markets [96]. The consistent aims of legislation are to govern, promote best practice, assess risk, and enhance consumer knowledge, such that, as the understanding of nanomaterials increases, authorities have the framework in place to identify and remediate any health or environmental risk. It is recommended in Rauscher, Rasmussen and Sokull-Klüttgen that still more research is required to address specific questions relating to regulation, with emphasis on the implementation of the definition of nanomaterials provided by the European Commission (EC,) enforcement of product labelling for the presence of nanomaterials, and the development of methods to test the safety of nanomaterials [96].

### 9.4. China

Although, a holistic regulation on nanotechnology is not present in China, there are organisations, funded by the government to conduct research on the environmental, health, and safety implications of nanotechnology. Cellular toxic effects of nanomaterials are researched by the National Centre for Nanoscience and Technology (NCNST) and the Chinese Academy of Sciences (CAS) [97], while toxicology studies on nanomaterials intended to be used in medicine are being led by the Chinese Academy of Medical Sciences, CAS, Beijing University, and other institutions [98]. In 2006, a Nanosafety Lab was set up by NCNST to study all aspects associated with nanotechnology; including the detection of nanoparticles, identifying nano-hazards, investigating the behaviours of nanoparticles in various environments, collecting data on the toxicity of nanoparticles, drafting regulatory frameworks for industry and research on nanotechnology and instituting standard procedures for safety assessment of nanomaterial [99]. Currently there are over 30 research organisations investigating the environmental and toxicological effects of nanoparticles and nanomaterials and establishing methods to recover nanoparticles from manufactured products in China [100].

### 9.5. International Bodies

The Organisation for Economic Cooperation and Development (OECD) launched the Working Party for Manufactured Nanomaterials (WPMN) in 2006 to reflect the rise in commercial applications of nanomaterials and to ensure that the approaches to associated hazards, exposure, and risk assessment for manufactured nanomaterials are high quality, science-based, and internationally harmonised [101]. The OECD, primarily through the subsidiary WPMN, is identified as being a key body in the governance of nanotechnology issues, with efforts complemented by the work of the OECD National Co-ordinators of the Test Guidelines Programme [102]. Through national and international research efforts, the WPMN have addressed and developed original questions identified in early stages of the party. However, the efforts made have been hampered due to a variety of issues encumbering the reporting process, such as the safeguarding of intellectual property rights.

In 2013, utilising the results gathered from the WPMN testing programme, related OECD subsidiaries, and the general development in understanding of nanomaterials independent of the WPMN, the OECD and member countries concluded that the current testing and assessing procedures of traditional chemicals are generally appropriate for assessing the safety of nanomaterials [102]. The general acceptance of this conclusion is predicated on the possibility of alteration to testing and assessing procedures based upon nanomaterial specificity. Through the Mutual Acceptance of Data OECD Council Decision instituted in 1981, all test data generated in any member countries are acceptable for the purpose of assessment and similar human health and environmental applications provided test data are generated according to the OECD Test Guidelines and Principles Good Laboratory Practice (GLP) [103,104].

The OECD efforts are supported by the International Organisation for Standardisation (ISO), which established the technical committee ISO/TC 229 in 2005. The technical committee was established to address standardisation within the field of nanotechnology. The scope of works includes the understanding and control of both matter and processes in the nanometre range where size dependent phenomena generally enable new applications, and the utilisation of these phenomena to improve materials, devices, and systems [105]. ISO/TC 229 has thus provided a standard framework for terminology, nomenclature, metrology, instrumentation, requirements of reference materials, testing methodology, modelling, simulation and health, safety, and environmental practices [96].

## 10. Discussion and Conclusions

Nanoscale materials offer a new range of possibilities in a multitude of fields, with applications widely varying and ever increasing. As such, many industries have embraced the enhancements that nanoparticles can provide, progressing industry specific products. This is seen in a wide variety of applications, through the construction field with nanoparticle enriched recycled materials with similar mechanical properties as fresh material, drug delivery, and antibacterial attributes in clothing. In a causal relationship, the quantity of demand and supply have increased to match the trend in nanoscale material utilisation, vastly increasing the production rates from previous years. As the rise of nanoparticles transpired so quickly, regulation regarding the health, safety, and environmental considerations of production, product application and end of life disposal has struggled to stay astride of the industry. It is integral that the necessary considerations be made when considering the long-term effects that the nanoparticle industry may give rise to. This is evidenced in studies demonstrating the toxicity of materials previously thought to be mammalian nontoxic. This is attributable to the minute size and formation of the particles, which alter the interactions with biological systems. Nanoparticle production and utilisation demand oversight and regulation prior to widespread introduction to avoid any detrimental effects that may not have come to light due to the relative youth of nanoparticles as a commercially viable production.

Nanoparticles have been identified as the next frontier of development in a variety of fields, with applications that yield promising results previously unforeseen. This is exemplified by the EU’s identification of nanoparticles as being key enabling technology, implying the importance for both technological and economic advancement within the EU. Due to the inevitability of nanoparticle utilisation, regulation and oversight is key to ensure that the growth of this industry is socially and environmentally beneficial. To achieve this goal, a responsible body for legislation and regulation is required to set a unified standard such that obscurity regarding best practice may be avoided. The OECD through a working party have established an international body dedicated to nanotechnology and industry, working through participating nations and providing an international standard. This is complemented by the ISO-established ISO/TC 229, the technical committee responsible for the standardisation of the industry. Through provision of nomenclature, terminology, best practice and reference requirements, a framework for Australian legislation and regulation may be developed to more broadly consider nanoparticles.

Major concerns are raised regarding the introduction of engineered or incidental nanoparticles into the environment, through a multitude of routes. It is integral that nanoparticle channels from industry to the environment be considered and everything practicable be done to remediate emissions. Due to a lack of legislation governing the specific release of nanoparticles into the environment, there is no framework or requirement by the industry to meet nanoparticle release goals. There needs to be a clearer understanding of the roles that nanoparticles play in the environment and the negative effects of exposure to these particles in these environments. Thus, a further review of the existing literature and more intensive research into biological and environmental interactions with nanoparticles is crucial. It is recommended that an industry-based review of current practices be undertaken to assess the applications of nanoparticles in specific industries to address, more specifically, industry resolutions and ensure that regulation accurately addresses specialised issues.

Conveyance of information regarding nanoparticles is required to ensure that consumers are aware of the constituent materials with which they are dealing such that they may make an informed decision. This information is currently unavailable or intermittent, with a lack of consistency. With the rise of nanoparticles, public awareness will increase. As such, it is important that the relationship be transparent between the consumer and the product, thereby reducing the likelihood of a negative public backlash.

Further to considering the intentional engineering of nanoparticles, consideration must be given to historic anthropological activities that give rise to unaddressed toxic nanoparticles. Of key consideration, nanoparticles generated through the use of common means of transportation that inevitably entail elevated shear force, heat, and velocity between materials, resulting in large deposition of nanoscale materials. The generation of this material may be malign in the environments that nanoparticles eventual deposit, as ultrafine particles of road rubber and brake pads can be highly toxic, adversely affecting the biomes to which they are exposed. Analysis of the current generation trends and total surplus of road rubber and brake wear fine–ultrafine material must be undertaken to quantify the effects of automotive centric means of transport. As growth in the population occurs internationally, a similar rise in the utilisation of automotive vehicles will be seen, which, in turn, will drive the generation of incidental particles that in the current framework lack a remediation methodology. It is recommended that further research be done on the cumulative damage resulting from the indiscriminate use of automotive vehicles and feasible solutions to address toxic nanoparticle deposition in waterways. Current research is being undertaken to develop viable biodegradable tyre products, a step to mitigating the effects of nanoparticle generation; however, other alternatives must be considered. Highlighted in the assessment of incidental nanoparticle generation is the need for easily accessible, wide reaching, and reliable public transport to reduce reliance on roads and automotive vehicles in an attempt to reduce the annual production of tyre wear products.

Nanoparticles as an industry provide a new frontier of production, enabling technological, health, and environmental advancements. To ensure the beneficial implementation of these pioneering products, further research and monitoring is necessary concerning the health of workers regularly exposed to nanoparticles, the quantities of nanoparticles released to the environment annually, pre-emptive technologies that reduce negative incidental nanoparticle production, and the effect of nanoparticles in biological systems.

## Figures and Tables

**Table 1:** Nanomaterials used in construction materials.

Application	Area	Nanoparticle Type	Major Applications
Construction	Concrete	Silica nanoparticles	• Reinforcement in mechanical strength • Rapid hydration
	Concrete	Titania nanoparticles	• Increased degree of hydration • Self-cleaning
	Concrete	Carbon nanotubes	• Mechanical durability • Crack prevention
	Asphalt concrete Timber	Aluminium oxide nanoparticles	• Increased serviceability
	Bricks mortar	Clay nanoparticles	• Increased compressive strength • Increased surface roughness
	Concrete	Iron oxide nanoparticles	• Increased compressive strength • Abrasion-resistant
	Steel	Copper nanoparticles	• Weldability • Corrosion resistance; Formability
	Asphalt concrete	Zycosoil	• Increased fatigue life • Higher compaction

**Table 2:** Process of engineered particle synthesis and particle type. Table produced utilising data supplied in Engineered Nanoparticles, Institut de recherche Robert-Sauvé en santé et en sécurité du travail (IRSST) [23].

Approach	Process	Nanoparticle Type
Chemical process	Vapour phase reactions	- Carbides - Nitrides - Oxides - Metallic alloys
	Reactions and precipitations in liquid media	- Metals - Oxides
	Reactions in solid media	- Metals - Oxides
	Supercritical fluids with chemical reactions	- Metals - Oxides - Nitrides
	Sol-gel techniques	- Oxides
Physical process	Evaporation or condensation under partial or inert pressure	- Iron (Fe) - Nickel (Ni) - Cobalt (Co) - Copper (Cu) - Aluminium (Al) - Palladium (Pd) - Platinum (Pt) - Oxides
	Laser pyrolysis	- Silicon (Si) - Silicon carbide (SiC) - Silicon carbonitride (SiCN) - Silaketenylidene (SiCO) - Silicon nitride (Si_3_N_4_) - Titanium carbide (TiC) - Titanium dioxide (TiO_2_) - Fullerenes - Carbonated soot - Metal oxides
	Plasma synthesis	- Metal oxides
	Combustion	- Metal oxides
	Ionic or electronic irradiation	- Production of nanopores - Nanostructures immobilised in matrix
	Mechanical activation of powder metallurgy	- Ceramics - Metallics - Metal oxides - Polymers - Semiconductors
	Consolidation and densification	- Varied
	Deformation via torsion, lamination or friction	- Metal oxides

**Table 3:** Health implications of nanoparticles to the human body.

Nanoparticle Type	Affected Cell/Organ/System	References
Silver nanoparticle (Ag NP)	- Immune system - Lungs - Liver - Brain - Carcinogenesis - Vascular system - Reproductive organs - Fibroblast	[69] [69,70] [70] [70] [70] [70] [70] [70]
Titanium dioxide (TiO_2_)	- Inflammation in lungs - DNA damage - Metabolic changes - Carcinogenesis - Cell death	[71,72] [71,73] [71] [71] [74]
Zinc oxide Nanoparticles (ZnO NP)	- Cell proliferation	[75]
Iron oxide (Fe_3_O_4_)	- Oxidative DNA damage	[73]
Copper zinc ferrite (CuZnFe_2_O_4_)	- DNA damage - Oxidative DNA damage	[73] [73]
Carbon nanotubes (CNT)	- DNA damage - Oxidative stress - Inflammation	[73] [76] [76]
Copper oxide (CuO)	- DNA damage - Oxidative DNA damage	[73] [73]
Silica nanoparticles (SiO_2_)	- Bronchoalveolar carcinoma-derived cells	[77]
